# Transcriptional Changes in CD16+ Monocytes May Contribute to the Pathogenesis of COVID-19

**DOI:** 10.3389/fimmu.2021.665773

**Published:** 2021-05-24

**Authors:** Vanessa Chilunda, Pablo Martinez-Aguado, Li C. Xia, Laura Cheney, Aniella Murphy, Veronica Veksler, Vanessa Ruiz, Tina M. Calderon, Joan W. Berman

**Affiliations:** ^1^ Department of Pathology, Albert Einstein College of Medicine, Bronx, NY, United States; ^2^ Department of Epidemiology and Public Health, Division of Biostatistics, Albert Einstein College of Medicine, Bronx, NY, United States; ^3^ Department of Medicine, Division of Infectious Diseases, Montefiore Medical Center and Albert Einstein College of Medicine, Bronx, NY, United States; ^4^ Department of Microbiology and Immunology, Albert Einstein College of Medicine, Bronx, NY, United States

**Keywords:** non-classical monocytes, single-cell transcriptomics, inflammation, cell movement, SARS-CoV-2

## Abstract

The COVID-19 pandemic has caused more than three million deaths globally. The severity of the disease is characterized, in part, by a dysregulated immune response. CD16+ monocytes are innate immune cells involved in inflammatory responses to viral infections, and tissue repair, among other functions. We characterized the transcriptional changes in CD16+ monocytes from PBMC of people with COVID-19, and from healthy individuals using publicly available single cell RNA sequencing data. CD16+ monocytes from people with COVID-19 compared to those from healthy individuals expressed transcriptional changes indicative of increased cell activation, and induction of a migratory phenotype. We also analyzed COVID-19 cases based on severity of the disease and found that mild cases were characterized by upregulation of interferon response and MHC class II related genes, whereas the severe cases had dysregulated expression of mitochondrial and antigen presentation genes, and upregulated inflammatory, cell movement, and apoptotic gene signatures. These results suggest that CD16+ monocytes in people with COVID-19 contribute to a dysregulated host response characterized by decreased antigen presentation, and an elevated inflammatory response with increased monocytic infiltration into tissues. Our results show that there are transcriptomic changes in CD16+ monocytes that may impact the functions of these cells, contributing to the pathogenesis and severity of COVID-19.

## Introduction

Coronavirus disease 2019 (COVID-19) is a global pandemic that has caused more than three million deaths worldwide since December 2019 (1). COVID-19, caused by Severe Acute Respiratory Syndrome Coronavirus 2 (SARS-CoV-2), predominantly impacts the respiratory system, but also the cardiovascular, hepatic, renal, and neurologic systems. The majority of individuals infected with SARS-CoV-2 can be asymptomatic or have mild symptoms including fever, myalgias, upper and/or lower respiratory tract symptoms, nausea, anorexia, and in some people, anosmia and/or dysgeusia (2). Some people with mild illness experience debilitating symptoms that can last for months. Approximately 15% of people with COVID-19 develop severe respiratory illness characterized by hypoxemia and hypoxia, resulting, in part, from Acute Respiratory Distress Syndrome (ARDS) and ventilation-perfusion mismatch (3). This is typically accompanied by multiorgan system failure. Severe disease leads to death in approximately 1-5% of affected people (3, 4).

Virus-host cell interactions and host immune responses are believed to be major factors contributing to COVID-19. However, these have not been fully characterized. Disease severity has been associated with an altered immune response, including highly elevated levels of inflammatory cytokines, lymphopenia, and significant mononuclear cell infiltration into organs (5–8). Understanding the host response that contributes to the pathophysiology of COVID-19 will further the development of therapeutic interventions against SARS-CoV-2 infection and its damaging consequences.

Monocytes play key roles in innate immunity. While they may not be permissive to SARS-CoV-2 replication, some reports suggest that these cells are important in COVID-19 pathogenesis. Monocytes are myeloid cells predominantly derived from the bone marrow, and comprise approximately 5-10% of peripheral blood mononuclear cells (PBMC). They play a crucial role in immune responses to viral infections by performing important functions that include phagocytosis, cytokine production, and antigen presentation. Monocytes are characterized by their surface markers: classical monocytes express high levels of CD14, the LPS co-receptor, and intermediate and non-classical monocytes express both CD14 and CD16, the FcγIII receptor (CD16+) (9–11). Peripheral blood CD16+ monocytes can be activated to produce an inflammatory response when foreign antigens are encountered, and also migrate into tissues where they can differentiate into macrophages (12). Another important function of CD16+ monocytes is patrolling the endothelial cell surface, ensuring that the balance between vascular injury and repair is maintained, and coordinating inflammatory responses in the presence of pathogens or foreign antigens (13). Some studies suggest that SARS-CoV-2 can infect endothelial cells, causing local inflammation (6, 14). This inflammation results in recruitment of CD16+ monocytes that can contribute to hyperinflammation and hypercoagulability, possibly leading to the multiorgan failure observed in many of the severe cases of COVID-19 (15–17).

There are reports of changes in the number of CD16+ monocytes in the PBMC from people with COVID-19. Some groups showed an increase in number of CD16+ monocytes from people with mild disease compared to healthy controls, but decreased numbers from people with severe disease (18–21). This decrease may be due to their migration into tissues, as studies showed that CD16+ monocytes are enriched within the lung tissue of some people with severe disease (19, 20). Other studies also reported increased numbers of inflammatory monocyte derived macrophages in bronchoalveolar lavage fluid from people with severe disease compared to those with mild disease, suggesting that peripheral blood monocytes migrate into lung tissue in people with severe COVID-19 (22, 23). These studies indicate that monocytes are recruited into tissues, where they can differentiate into macrophages and contribute to COVID-19 immunopathogenesis. While these studies showed there are changes in the numbers of circulating and infiltrated CD16+ monocytes in SARS-CoV-2 infection, little is known of how their biology contributes to COVID-19. It is also unclear whether specific subpopulations of CD16+ monocytes can directly contribute to the disease.

To characterize the contribution of these cells to SARS-CoV-2 pathogenesis, we performed an integrative single cell RNA sequencing (scRNAseq) analysis of CD16+ monocytes obtained from publicly available data of PBMC from healthy people and those with COVID-19. We compared CD16+ monocytes from people with COVID-19 to those from healthy controls, and found increased inflammation and interferon response related genes, suggesting that CD16+ monocytes contribute to the immune response against SARS-CoV-2. The expression of cell movement associated genes was also increased in these cells, indicating that they may be recruited into tissues where they contribute to disease processes. To characterize the specific contribution of CD16+ monocyte responses to different clinical presentations of disease, we compared mild or severe cases to healthy controls. While mild cases had upregulated interferon response genes, severe cases were characterized by increased inflammatory and cell movement gene signatures. When we also compared the transcriptome profiles of CD16+ monocytes from people with severe disease to those from people with mild COVID-19, we found that severe cases had dysregulated genes involved in protein synthesis, oxidative phosphorylation, and apoptosis related functions. Thus, this study characterizes CD16+ monocyte transcriptome changes that may impact the functional properties of these cells during COVID-19, providing an understanding of how these changes may contribute to pathogenesis and disease severity.

## Methods

We integrated and analyzed published single-cell RNA sequencing (scRNA-seq) data to study gene expression of CD16+ monocytes, and to examine the possible contributions of these cells and their specific subsets to the pathogenesis of COVID-19. The data collection, extraction, integration, and analysis steps are described below.

### Data Sources

We identified five published studies with publicly available single-cell RNA sequencing data of PBMC from healthy people and from people with COVID-19 (24–28). We downloaded these data from the Gene Expression Omnibus (GEO) database with the accession numbers GSE150728 and GSE149689, the European Genome-phenome Archive (EGA) under access number EGAS00001004571 and from the FASTGenomics website (https://beta.fastgenomics.org/p/565003). We included a publicly available healthy control sample downloaded from the 10X Genomics official website (https://support.10xgenomics.com/single-cell-gene-expression/datasets/3.1.0/5k_pbmc_NGSC3_aggr).

### Quality Control and CD16+ Monocyte Extraction

**Table 1** summarizes the datasets and status of the participants included in this study. Disease severity was classified according to the original publications, except for the data obtained from the Bernandez et. al., (27). For this study, we reclassified participants as mild or severe by their maximum 11-point World Health Organization (WHO) Ordinal Scale for Clinical Improvement, and the amount of time in which participants were symptomatic prior to the first PBMC sampling. We did not include all participants of this study as some samples were obtained from convalescing participants, or mild or severe disease status could not be deduced with the information provided. Sample-wise unique molecular identifier (UMI) count matrices from these studies were combined and input into the Seurat package (version 3.2.2) on R (version 4.0.2) for further quality control (QC) and data extraction.

**Table 1:** Sample name, origin of the data, age, gender, and disease severity of the individuals from whom scRNAseq data were derived after exclusion of donors, as described in Methods.

Sample name	Paper	Age group	Sex	Disease severity
Cov2 Sample 1	(23), *Nat. Med*	60-69	M	COVID-19, severe
Cov2 Sample 2	(23), *Nat. Med*	30-39	M	COVID-19, severe
Cov2 Sample 7	(23), *Nat. Med*	20-29	M	COVID-19, severe
Healthy Sample 3	(23), *Nat. Med*	40-49	M	Healthy
Healthy Sample 4	(23), *Nat. Med*	40-49	M	Healthy
Cov2 Sample 1b	(24), *Sci. Immunol*	60-69	M	COVID-19, severe
Cov2 Sample 4b	(24), *Sci. Immunol*	40-49	M	COVID-19, mild
Cov2 Sample 5b	(24), *Sci. Immunol*	30-39	F	COVID-19, mild
Cov2 Sample 7b	(24), *Sci. Immunol*	60-69	M	COVID-19, mild
Cov2 Sample 8b	(24), *Sci. Immunol*	60-69	F	COVID-19, mild
Healthy Sample 1b	(24), *Sci. Immunol*	60-69	F	Healthy
Healthy Sample 2b	(24), *Sci. Immunol*	50-59	F	Healthy
Healthy Sample 3b	(24), *Sci. Immunol*	60-69	F	Healthy
Healthy Sample 4b	(24), *Sci. Immunol*	60-69	M	Healthy
Cov2 Sample 1c	(25), *Cell*, (Cohort 1)	20-29	M	COVID-19, mild
Cov2 Sample 2c	(25), *Cell*, (Cohort 1)	40-49	M	COVID-19, mild
Cov2 Sample 3c	(25) , *Cell*, (Cohort 1)	70-79	M	COVID-19, mild
Cov2 Sample 4c	(25), *Cell*, (Cohort 1)	70-79	F	COVID-19, mild
Cov2 Sample 5c	(25), *Cell*, (Cohort 1)	60-69	M	COVID-19, severe
Healthy Sample 1c	(27), *Nat Med*	N/A	N/A	Healthy
Healthy Sample 2c	(27), *Nat Med*	N/A	N/A	Healthy
Healthy Sample 10c	(27), *Nat Med*	N/A	N/A	Healthy
Healthy Sample 11c	(27), *Nat Med*	N/A	N/A	Healthy
Healthy Sample 12c	(27), *Nat Med*	N/A	N/A	Healthy
Healthy Sample 13c	(27), *Nat Med*	N/A	N/A	Healthy
Healthy Sample 15c	(27), *Nat Med*	N/A	N/A	Healthy
Healthy Sample 16c	(27), *Nat Med*	N/A	N/A	Healthy
Healthy Sample 17c	(27), *Nat Med*	N/A	N/A	Healthy
Cov2 Sample 1d	(25), *Cell*, (Cohort 2)	80-89	M	COVID-19, mild
Cov2 Sample 2d	(25), *Cell*, (Cohort 2)	60-69	M	COVID-19, mild
Cov2 Sample 2e_TA	(26), *Immunity*	50-59	M	Healthy
Cov2 Sample 4e_TB	(26), *Immunity*	60-69	M	Healthy
Cov2 Sample 5e_TA2	(26), *Immunity*	40-49	M	Healthy
Cov2 Sample 8e_TA	(26), *Immunity*	60-69	F	Healthy
Healthy Sample 2e	(26), *Immunity*	N/A	N/A	Healthy
Healthy Sample 10X	10X Genomics website	N/A	N/A	Healthy

For each donor, we first visualized the QC metrics and discarded cells that had less than 200 expressed genes as well as cells that had high mitochondria expression, which ranged from 15-25% of all expressed genes. We used the SCTransform function to normalize the dataset using a regularized negative binomial model, which was adjusted for mitochondrial mapping percentage. For data visualization, we performed dimensionality reduction using the Principal Component Analysis (PCA) and the Unifold Manifold Approximation and Projection (UMAP) embedding. We then used the FindClusters function using a nearest neighbor search (SNN) with a resolution of 0.5 to cluster the cells.

To identify specific cell types, we applied the FindAllMarkers function to identify cluster-wise differentially expressed genes using p-value < 0.01 after Bonferroni correction. We identified CD16+ monocyte clusters based on the high expression of their canonical markers *FCGR3A* and *MS4A7*. The subset function was applied to extract the identified CD16+ monocyte clusters from each dataset. We next merged the extracted CD16+ monocytes from all studies and performed an additional quality control to remove any contaminant T cells and NK cells from the previously identified monocytes, by excluding those cells that expressed *B3GAT1*, and *CD3E*, respectively. After this process, we obtained the final subsets of CD16+ monocytes. We only included donors (n=36) who had substantial and comparable number of CD16+ monocytes (i.e. >50 cells) for further analysis. The samples analyzed in this study include 6 severe, 13 mild and 17 healthy donors.

### CD16+ Monocyte Integration

To integrate the data, we first performed SCTransform normalization on the merged dataset as described above. Next, we used the intersection of genes that occurred in all datasets as the features to integrate. For that, we applied the FindIntegrationAnchors function that uses canonical correlation analysis to identify anchors between the datasets. Next, using the identified anchors, we performed data integration by using the IntegrateData function, setting 10X genomics dataset as the reference sample.

### CD16+ Monocyte Sub-Clustering

After completing the integration step, we performed dimensionality reduction using the top 10 principal components with a resolution of 0.4 to obtain CD16+ monocyte sub-clusters. These sub-clusters were visualized using UMAP plots.

### Differential Gene Expression Analysis

We used FindMarkers function with Wilcoxon Rank sum test to obtain a list of differentially expressed genes (DEG) in CD16+ monocytes from healthy people and from those with COVID-19. First, we determined global DEG between CD16+ monocytes from healthy and COVID-19 samples. We then compared gene expression between healthy and COVID-19 samples within the four different CD16+ monocyte sub-clusters. We also obtained DEG in CD16+ monocytes based on the severity of COVID-19, which was classified as mild or severe, as described above. We performed pairwise comparisons between severe and mild; severe and healthy; and mild and healthy cases. Genes that had Bonferroni corrected p-value <0.01, and genes with >0.25 log fold changes were considered significantly different. Top ranked genes based on adjusted p-value were extracted for visualization.

### Visualization of Differentially Expressed Genes

To visualize the expression of genes between different conditions or clusters, we used FeaturePlot, VlnPlot, DoHeatMap, and DotPlot functions.

### Pathway Analysis

The lists of significant genes were analyzed using Ingenuity Pathway Analysis (IPA) software (Qiaqen). We used a cut-off Z-score of either >2 or <-2 to determine significantly changed pathways, or diseases and functions.

### Data Validation

To validate our results, we performed a separate analysis of a recently published dataset with the GEO accession number GSE158055. We extracted CD16+ monocytes, performed integration, and differential gene expression analysis as described above. The samples analyzed from this validation cohort include 14 healthy, 10 mild and 8 severe donors. Due to the large number of CD16+ monocytes in this dataset, we did not include it in our analyses to avoid introducing bias during integration analysis.

## Results

### CD16+ Monocytes From People With COVID-19 Compared to Healthy Controls Have Upregulation of Inflammatory and Interferon Response Genes

**Figure 1:**
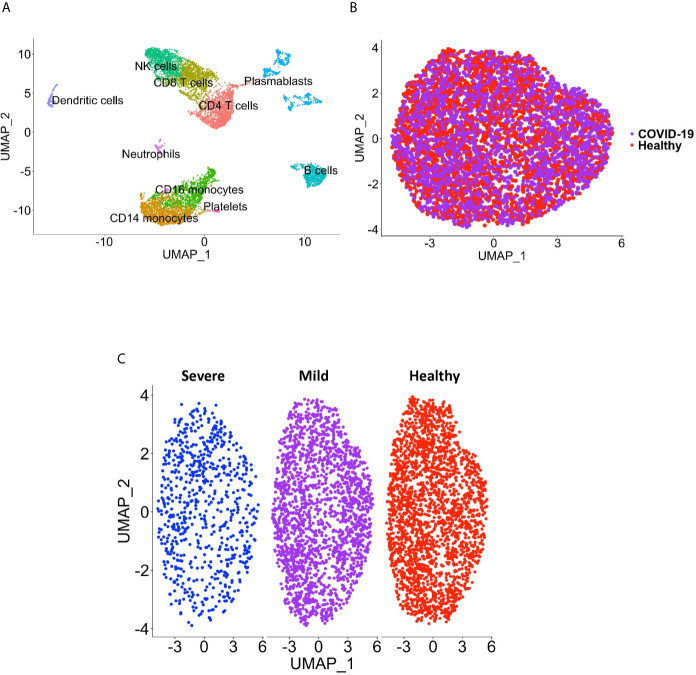
CD16+ monocyte identification and integration analyses. **(A)** A representative UMAP plot showing identification of CD16+ monocytes based on unsupervised clustering. **(B)** A UMAP plot after integration of all donor datasets indicate no technical batch effects. COVID-19 samples are shown in purple, and healthy samples in red. **(C)** UMAP plot showing CD16+ monocytes from severe (blue), mild (purple) and healthy (red) cases.

**Figure 2:**
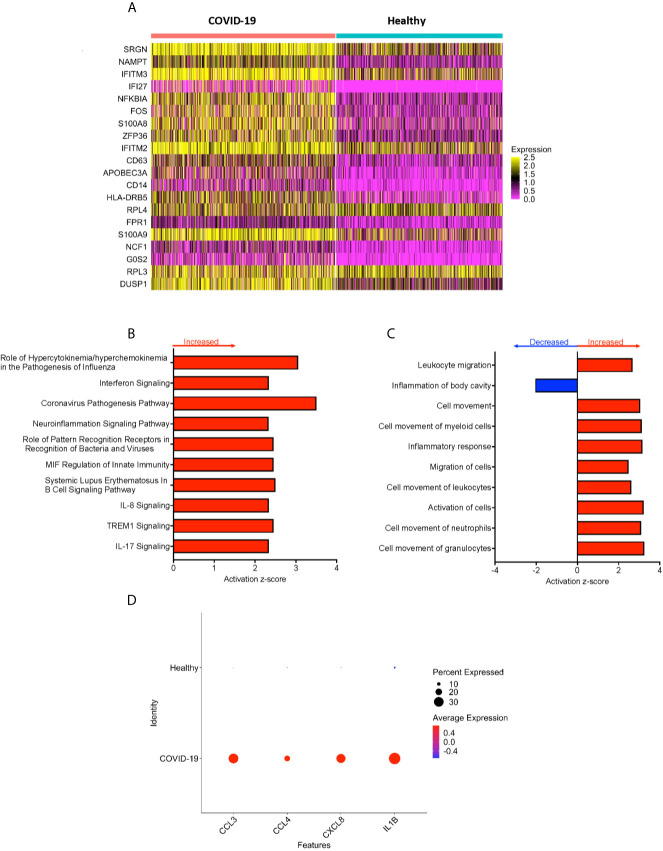
CD16+ monocytes from COVID-19 samples compared to healthy controls have dysregulated genes related to inflammation and metabolic pathways. **(A)** A heatmap of the top 20 DEG between CD16+ monocytes from people with COVID-19 and healthy individuals. Each column depicts cells from COVID-19 cases or healthy individuals, and rows indicate the top DEG. The DEG heatmap is colored by normalized gene expression levels. Yellow represents higher expression, and pink represents lower expression. **(B, C)** IPA of DEG showing the top 10 **(B)** cellular pathways and **(C)** diseases and functions predicted to be increased (red) or decreased (blue) in CD16+ monocytes from COVID-19 cases compared to healthy controls. **(D)** Dot plot of some DEG involved in inflammation between COVID-19 and healthy controls, represented by a color scale showing lower (blue) to higher (red) gene expression. The size of the dot represents the percentage of cells expressing each specific gene.

Peripheral blood CD16+ monocytes are important in the host response to viral infections. COVID-19 is characterized by an altered immune and inflammatory response. We examined changes in the transcriptomic profiles of CD16+ monocytes to study potential contributions of these cells to SARS-CoV-2 pathogenesis. We analyzed specific changes in the single cell transcriptome profile of CD16+ monocytes from PBMC from people with COVID-19 and healthy individuals using publicly available scRNAseq data. The metadata of these groups are shown in **Table 1**. The data were downloaded from publicly available sources as detailed in the Methods. For each individual dataset, we performed dimensionality reduction algorithm using Principal Component Analysis (PCA) and Unifold Manifold Approximation and Projection (UMAP) embedding to identify CD16+ monocyte clusters (**Figure 1A**). CD16+ monocytes were identified and extracted based on high expression of *FCGR3A* and *MS4A7*, which are canonical markers of these cells. After CD16+ monocytes were extracted and identified from each individual, cells were normalized, integrated, and dimensionally reduced. We did not observe any batch effects after the data integration process for the extracted CD16+ monocytes (**Figure 1B**). UMAP visualization of cells according to disease severity demonstrated that the severe COVID-19 group had a lower number of CD16+ monocytes compared to the group with mild COVID-19, and also as compared to healthy controls (**Figure 1C**). This is consistent with previous reports of decreased CD16+ monocytes in the peripheral blood of people with severe COVID-19 (18–21). To examine transcriptional changes in CD16+ monocytes in the context of SARS-CoV-2 pathogenesis, we identified DEG in cells from people with COVID-19 compared to those from healthy controls (**Supplementary Table 1**). The gene expression heatmap showed upregulation of genes involved in the antiviral interferon response (*IFITM*, *IFIT* and *IFI* family genes), and those for the S100 calcium and zinc binding protein family (*S100A8* and *S100A9*) that are involved in inflammatory processes (29) (**Supplementary Table 1** and **Figure 2A**). The increase in interferon related genes is consistent with other studies that report similar results in monocytes from COVID-19 cases (24).

To analyze the potential biological significance of the transcriptional changes in CD16+ monocytes from people with COVID-19, we performed IPA of DEG. We found upregulated pathways related to interferon signaling and increased cytokine and chemokine production (**Figure 2B**). There were predicted functions related to cell movement and inflammatory responses (**Figure 2C**). Compared to healthy controls, CD16+ monocytes from people with COVID-19 had significantly upregulated expression of genes involved in inflammation including cytokines and chemokines, such as *IL-1β*, CXCL8, *CCL3* (*MIP-1α*), and *CCL4* (*MIP-1β*) (**Figure 2D**). The large number of DEG associated with inflammatory pathways suggest that CD16+ monocytes contribute to the altered immune phenotype observed in COVID-19.

### The CD16+ Monocyte Subpopulation Distribution Is Not Changed in People With COVID-19 Compared to Healthy Donors

**Figure 3:**
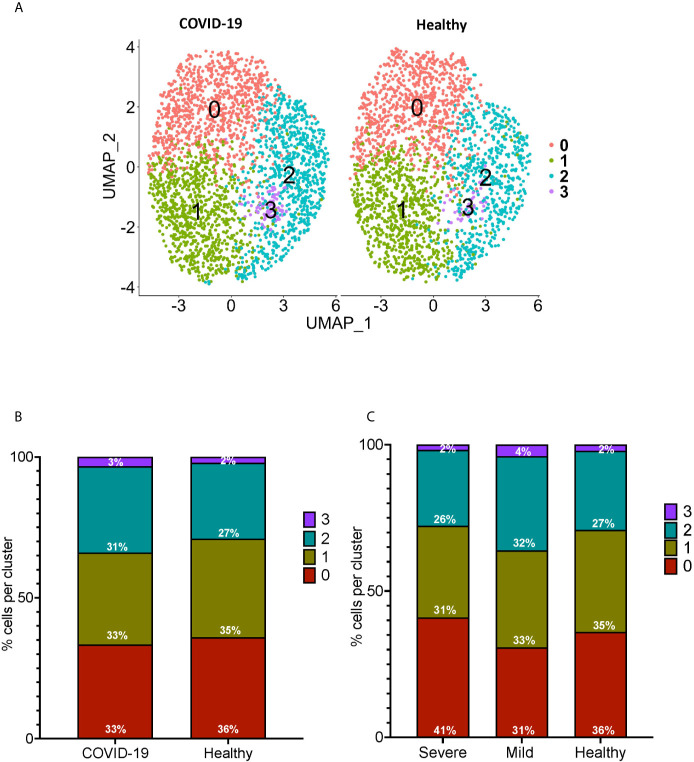
Subclustering analysis show similar subpopulations of CD16+ monocytes from COVID-19 and healthy controls. **(A)** UMAP plot showing subclustering of CD16+ monocytes from COVID-19 cases (left) resulted in similar subpopulations to those found in healthy controls (right). **(B)** There are similar percentages of cells within the CD16+ subpopulations in COVID-19 samples compared to healthy samples. **(C)** There are similar percentages of cells within the CD16+ subpopulations in severe and mild COVID-19 samples compared to healthy samples.

We performed sub-clustering analysis of the CD16+ monocytes from COVID-19 cases and healthy controls to identify specific transcriptome signatures among CD16+ monocytes. We identified four subpopulations of CD16+ monocytes and found no difference in the distribution of sub-clusters of these cells from COVID-19 cases compared to healthy controls (**Figure 3A**). We quantified the proportion of CD16+ monocytes in each of the subpopulations for both the COVID-19 cases and healthy controls (**Figure 3B**). COVID-19 cases had a similar total percentage of cells in each cluster as compared to healthy controls (**Figure 3B**). To characterize the distribution and significance of the subpopulations to COVID-19, we also quantified the percentage of cells based on disease severity, and found similar proportions of cells within each subcluster (**Figure 3C**). We performed DEG analysis to determine changes between cells from COVID-19 cases and healthy controls within each specific subpopulation (**Supplementary Table 2**). We found that all subpopulations of CD16+ monocytes had similar DEG (**Supplementary Table 2**). IPA demonstrated that cells from all the clusters had increased predicted functions related to cell movement, inflammation, and cell activation (data not shown) suggesting that all subpopulations of CD16+ monocytes contribute similarly to COVID-19 pathogenesis.

### CD16+ Monocytes From Mild COVID-19 Cases Compared to Those From Healthy Controls Express an Antiviral Immune Response Driven by Upregulated Interferon Stimulated Genes

**Figure 4:**
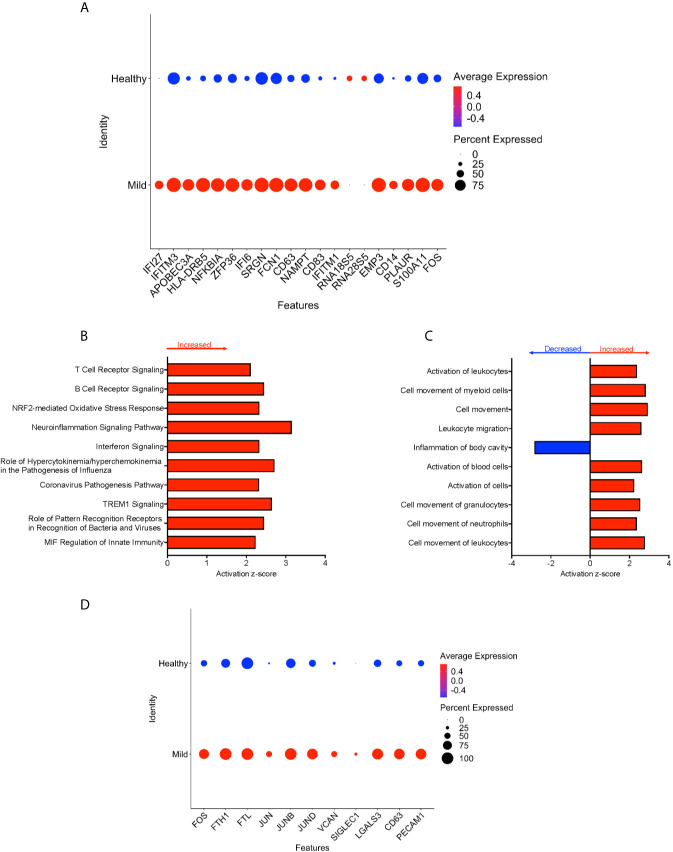
CD16+ monocytes from mild cases compared to healthy controls have a strong interferon response. **(A)** Dot plot of the top 20 DEG between mild cases and healthy controls, represented by a color scale showing lower (blue) to higher (red) gene expression. The size of the dot represents the percentage of cells expressing each specific gene. Top **(B)** pathways and **(C)** diseases and functions predicted to be increased (red) or decreased (blue) in mild cases compared to healthy controls. **(D)** Dot plot of some DEG involved in oxidative stress response and cell activation between mild cases and healthy controls, represented by a color scale showing lower (blue) to higher (red) gene expression. The size of the dot represents the percentage of cells expressing each specific gene.

We divided the COVID-19 donors into either mild or severe cases based on the published information, to characterize DEG that may contribute to disease severity. To characterize the genetic signature of CD16+ monocytes in people with mild COVID-19, we compared gene expression in CD16+ monocytes from mild cases to healthy individuals. We found a total of 702 significant DEG between these groups (**Supplementary Table 3**). DEG analysis and IPA showed a large number of upregulated interferon related genes (*IFI27, IFITM3*, *IFI6*, *IFITM1)* (**Figures 4A, B**). The IPA analysis also showed predicted increased cell movement and activation genes (*SIGLEC1*, *LGALS3*, *VCAN*) and higher expression of genes involved in oxidative stress response (*FOS*, *JUN*, *JUNB*) (**Figures 4B–D** and **Supplementary Table 3**).

### CD16+ Monocytes From People With Severe COVID-19 Compared to Those From Healthy Controls Have an Increased Inflammatory Signature Characterized by Mitochondria Gene Dysregulation and Increased Expression of Cell Movement and Migration Genes

**Figure 5:**
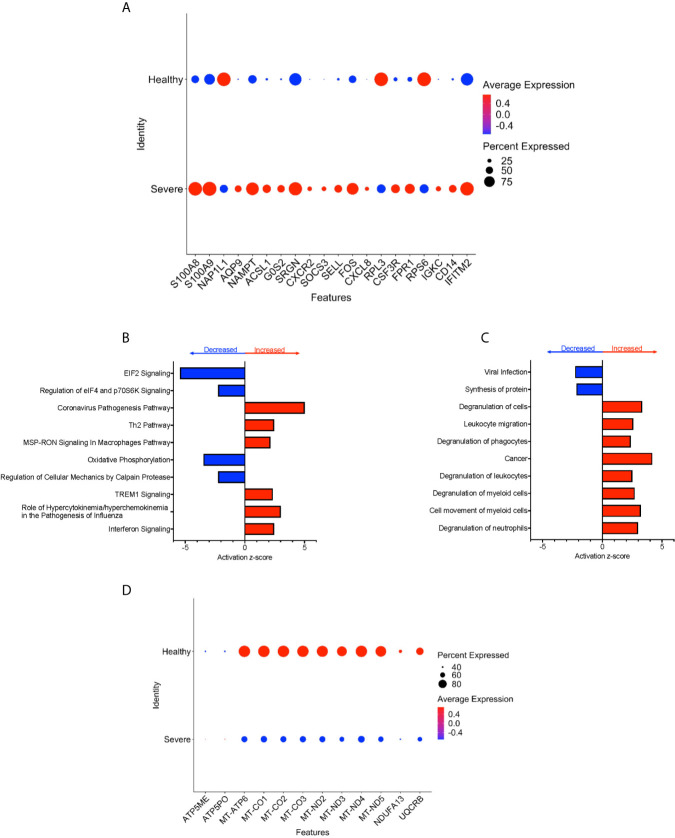
CD16+ monocytes from people with severe COVID-19 compared to healthy controls have upregulated inflammatory and cell movement genes. **(A)** Dot plot of the top 20 DEG between severe cases and healthy controls, represented by a color scale showing lower (blue) to higher (red) gene expression. The size of the dot represents the percentage of cells expressing each specific gene. Top **(B)** pathways and **(C)** diseases and functions predicted to be increased (red) or decreased (blue) in the severe cases compared to healthy controls. **(D)** Dot plot of DEG involved in oxidative phosphorylation between mild cases and healthy controls, represented by a color scale showing lower (blue) to higher (red) gene expression. The size of the dot represents the percentage of cells expressing each specific gene.

To characterize further the potential role of CD16+ monocytes in severe cases, we compared changes in the transcriptome of CD16+ monocytes from people with severe COVID-19 to those from healthy controls. We found a total of 702 significant DEG between these groups (**Supplementary Table 4**). DEG analysis showed highly expressed genes related to chemotaxis (*CXCR2, CSF3R*), cell adhesion (*SELL*), and inflammation (*S100A8*, *S100A9*) (**Figure 5A**).

We identified predicted dysregulated pathways related to metabolic homeostasis including oxidative phosphorylation, and EIF2 signaling pathways (**Figure 5B**). Consistent with changes in expression of chemotaxis, inflammation, and cell adhesion related genes, we found predicted altered functions related to cell movement and cell migration (**Figure 5C**).

We found several significantly downregulated DEG that encode for the mitochondrial electron transport chain complexes I and IV (*MT-CO1*, *MT-CO2*, *MT-CO3*, *MT-ND2*, *MT-ND3, MT-ND4*, *MT-ND5*), which are involved in oxidative phosphorylation (**Figures 5B, D** and **Supplementary Table 4**). These results suggest that reduced activity of these complexes can contribute to changes in oxidative phosphorylation, ROS accumulation, and mitochondrial damage leading to altered metabolic pathways and immune responses (30). Overall, these results indicate that CD16+ monocytes from people with severe COVID-19 showed significant metabolic changes, a marked inflammatory signature, and predicted increase in cell movement and migration compared to those from healthy controls.

### CD16+ Monocytes From Severe COVID-19 Cases Compared to Those From Mild Cases Have Dysregulated Protein Synthesis and Antigen Presentation Gene Expression, and Increased Expression of Apoptosis Related Genes

**Figure 6:**
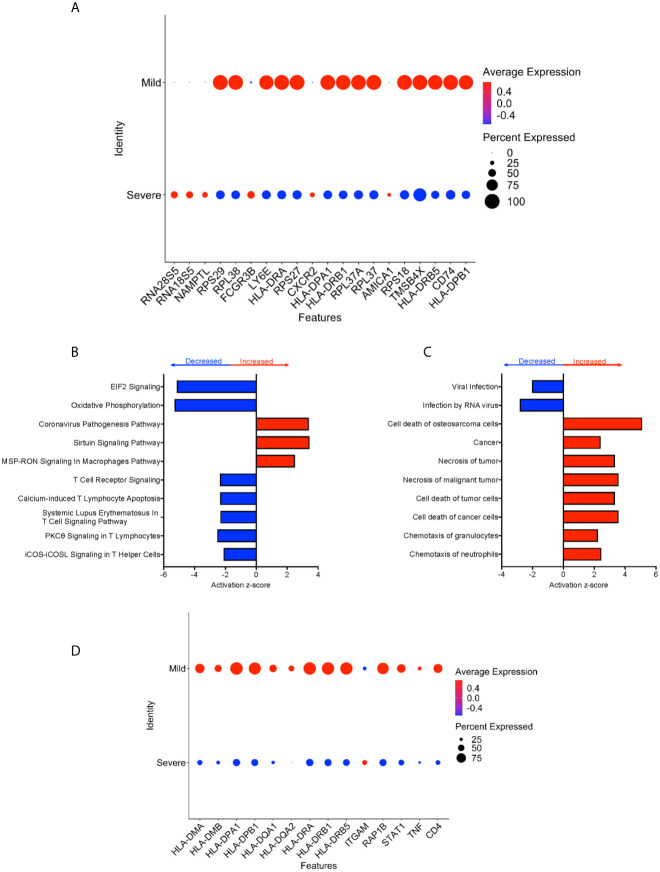
CD16+ monocytes from severe cases compared to those from mild cases have dysregulated protein synthesis, antigen presentation, and increased apoptosis related functions. **(A)** Dot plot of the top 20 DEG between severe and mild cases, represented by a color scale showing lower (blue) to higher (red) gene expression. The size of the dot represents the percentage of cells expressing each specific gene. Top **(B)** pathways and **(C)** diseases and functions predicted to be increased (red) or decreased (blue) in severe cases compared to mild cases. **(D)** Dot plot of some DEG involved in MSP-RON and T-cell receptor signaling pathways between mild cases and healthy controls, represented by a color scale showing lower (blue) to higher (red) gene expression. The size of the dot represents the percentage of cells expressing each specific gene.

We performed DEG analysis to compare gene expression between CD16+ monocytes from severe and mild cases. We found a total of 702 DEG, with 648 downregulated and 54 upregulated in severe compared to mild cases (**Supplementary Table 5**). There was significant downregulation of a large number of ribosomal genes including *RPS29*, *RPL38*, *RPS27*, *RPL37A*, and *RPS18*, suggesting inhibition of host cell translation initiation (**Figure 6A**). Expression of Human Leukocyte Antigen (HLA) class II genes, *HLA-DPA1*, *HLA-DPB1*, *HLA-DRA*, *HLA-DRB1* and *HLA-DRB5*, was significantly downregulated in severe compared to mild cases (**Figure 6A**). Decreased expression of these Major Histocompatibility Complex (MHC) class II genes in severe cases could interfere with the ability of CD16+ monocytes to present antigens. To identify cell pathways that may be differently affected in severe as compared to mild cases, IPA was performed. We found a significant predicted inhibition of EIF2 signaling and oxidative phosphorylation pathways in severe as compared to mild cases (**Figure 6B**). We analyzed the predicted affected functions for the DEG. There was predicted activation of several apoptosis functions in CD16+ monocytes from severe compared to mild COVID-19 cases (**Figure 6C**). This may result from endoplasmic reticulum stress induced in severe COVID-19 cases and is consistent with the observed predicted potent inhibition of the EIF2 and oxidative phosphorylation pathways (**Figure 6B**). We also found predicted activation of the macrophage stimulating-protein (MSP-RON) pathway in severe cases, with upregulation of the *ITGAM* gene, involved in the regulation of inflammatory responses (**Figures 6B, D**) (31). We also found a predicted downregulation of CD16+ monocyte genes involved in signaling with the T-cell receptor such as *CD4 and HLA* in severe cases (**Figure 6D**).

### Validation of Study Results After Analyses of Data From an Additional scRNAseq Study of PBMC From Individuals With COVID-19

**Figure 7:**
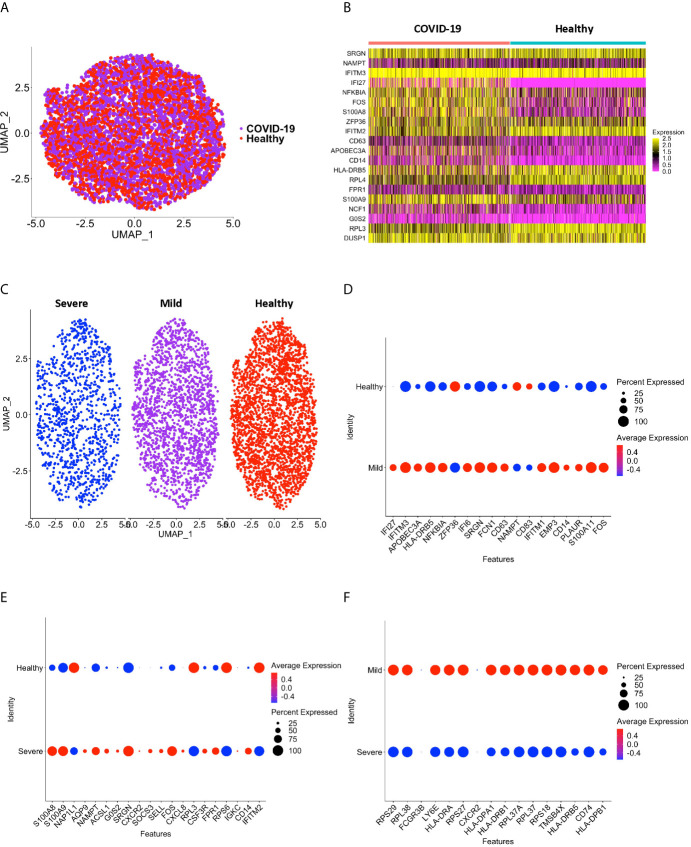
CD16+ monocytes from the (31) dataset have DEG between COVID-19 and healthy controls that are similar to our dataset. **(A)** A UMAP plot after integration of donors from the (31) dataset indicate no technical batch effects. COVID-19 samples are shown in purple, and healthy samples in red. **(B)** A heatmap validating DEG between CD16+ monocytes from people with COVID-19 and healthy individuals. Each column depicts cells from COVID-19 cases or healthy individuals, and rows indicate the top DEG. The DEG heatmap is colored by normalized gene expression levels. Yellow represents higher expression, and pink represents lower expression. **(C)** UMAP plot showing CD16+ monocytes from severe (blue), mild (purple) and healthy (red) cases. **(D)** Dot plot validating DEG that we found in this study between mild and healthy cases, represented by a color scale showing lower (blue) to higher (red) gene expression. The size of the dot represents the percentage of cells expressing each specific gene. **(E)** Dot plot validating DEG between severe and healthy cases, represented by a color scale showing lower (blue) to higher (red) gene expression. The size of the dot represents the percentage of cells expressing each specific gene. **(F)** Dot plot validating DEG between severe and mild cases, represented by a color scale showing lower (blue) to higher (red) gene expression. The size of the dot represents the percentage of cells expressing each specific gene.

To validate our results, we integrated scRNA-seq data from a different dataset obtained from a recently published study (**Supplementary Table 6**), and performed DEG analysis between CD16+ monocytes from individuals with COVID-19 and healthy controls (32) (**Figures 7A, B**). Using this dataset, we found that the DEG profile of CD16+ monocytes from people with COVID-19 as compared to those from healthy controls was similar to our obtained results described above. For example, inflammatory genes including *S100A8* and *S100A9* were upregulated in COVID-19 compared to healthy donors (**Figure 7B** and **Supplementary Table 7**). Although we found many other genes had similar differential gene expression in this data set compared to our study, there were a few genes that were not differentially expressed between COVID-19 and healthy donors in their study. Such genes include *IFITM2* and *IFITM3* (**Figure 7B** and **Supplementary Table 7**). This could be attributed, in part, to donor variability. The primary analyses in the current study were performed using data from COVID-19 cases originating from several countries, including the United States, Korea, and Germany. The recent study that was used to validate our findings was performed on COVID-19 cases entirely from different regions in China (32).

We also divided the cases in the validation study based on severity (**Figure 7C** and **Supplementary Table 7**). Similar genes as we found were upregulated in CD16+ monocytes from mild cases compared to healthy controls, including the interferon related genes *IFI6*, *IFI27*, and *IFITM3* (**Figure 7D** and **Supplementary Table 7**). When comparing severe and healthy cases, similar genes to what we described above related to chemotaxis (*CSF3R*), cell adhesion (*SELL*), and inflammation (*S100A8*, *S100A9*) were upregulated in severe cases (**Figure 7E** and **Supplementary Table 7**). In our analysis of their samples we also found downregulated genes, including the antigen presentation genes (*HLA-DPA1*, *HLA-DPB1*, *HLA-DRA*, *HLA-DRB1*, *HLA-DRB5*) in CD16+ monocytes from severe compared to mild cases (**Figure 7F** and **Supplementary Table 7**). IPA analysis also showed changes in pathways and functions similar to what we had observed (data not shown). These results validate our data, and further suggest a potential role of CD16+ monocytes in COVID-19 pathogenesis.

## Discussion

CD16+ monocytes are involved in the pathogenesis of viral and bacterial infections and inflammatory diseases, in which they produce cytokines, present antigens, regulate apoptosis, and phagocytose cell debris and foreign material (9, 11). Several studies suggest that these cells migrate from the peripheral circulation into lung tissue where they can differentiate into macrophages and continue to contribute to COVID-19 immunopathogenesis (18–21). In the present study, we characterized the transcriptome signature of CD16+ monocytes at a single cell-level to characterize the biology and immune response of these cells in people with and without SARS-CoV-2 infection, as well as to compare those with mild and severe COVID-19. The ultimate goal is to identify novel therapeutic strategies to diminish life-threatening outcomes of this disease.

Several studies underscored the importance of a type I interferon response as it relates to the severity of COVID-19 (33–35). In our analyses of CD16+ monocytes from COVID-19 cases compared to healthy controls, expression of many interferon stimulated genes (ISG) is increased. When COVID-19 cases were divided into mild and severe, we identified differences in the expression of ISG. *IFI6*, *ISG15*, *IFI27*, and *IFI35* were increased in mild, and decreased or not changed in severe COVID-19, compared to healthy controls. Some of these genes were also found to be differentially expressed in the validation dataset, underscoring the importance of this expression in COVID-19 pathogenesis. Other scRNAseq studies also reported dysregulated interferon responses in COVID-19. One study showed increased classical CD14+HLA-DR^hi^ monocytes with high *IFI6* and *ISG15* expression in mild COVID-19 cases when compared to healthy controls (26), similar to our findings. The type I interferon signaling pathway was reported to be increased in total peripheral monocytes in people with COVID-19 as determined by scRNAseq analysis with increased expression of genes including *IRF7* and *ISG15* (36). Another study also reported increased interferon signaling genes including *IFI44L*, *IFIT1*, *IFIT3* and *ISG15*, in CD16+ monocytes in COVID-19 cases compared to healthy controls (37). In our study, we report that *IRF7*, *ISG15*, *IFI44L*, *IFIT1*, and *IFIT3* gene expression is increased in CD16+ monocytes from people with mild COVID-19 compared to healthy controls. In people with severe disease, we found either no change or decreased expression of *IRF7*, *ISG15*, and *IFI44L* compared to healthy controls. In another study, single-cell transcriptomic analysis identified DEG indicative of a type I interferon signature in classical monocytes from people with mild COVID-19, while in people with severe COVID-19, this signature was decreased, and genes involved in production of ROS and nitric oxygen species (NOS) were increased (21). Similarly, we observed an induced interferon pathway in CD16+ monocytes from mild cases compared to healthy controls. Cumulatively, these data suggest that the increase in type I interferon signaling found in circulating CD16+ monocytes from mild cases may contribute to a strong antiviral response, whereas the dysregulated type I interferon response in these cells from severe cases may contribute to severity of the disease.

Recent studies showed that monocytes are important to COVID-19 inflammatory responses (38–40). We found increased expression of cytokine, chemokine, chemokine receptor, and adhesion molecule genes in CD16+ monocytes from people with COVID-19 compared to those from healthy individuals. A previous study that examined circulating cytokine levels from people with severe COVID-19 showed a decrease in IFN-γ and increased IL-6, CXCL8, IL-1β, and CCL2, as compared to healthy controls (41). This study concluded that the pattern of increased cytokines in COVID-19 suggests that there is an increase in innate inflammatory mediators associated with monocyte and neutrophil mobilization, rather than an upregulation in adaptive immune responses characterized by increased IFN-γ. Our data show increased cytokine and chemokine gene expression, including *CXCL8*, *IL-1β*, and *CCL3* (*MIP-1α*) in both mild and severe COVID-19, compared to healthy controls. These results suggest that CD16+ monocytes contribute to the inflammatory response observed in people with COVID-19. In addition, we found that CD16+ monocytes from people with severe COVID-19 had upregulated CXCL8 receptor genes, *CXCR1* and *CXCR2*, compared to mild cases. HIF-1α can induce upregulation of these three proteins, and hypoxia and viral infections have been shown to upregulate *CXCL8* in monocytes (42). These findings suggest that upregulation of *CXCL8* and its receptors, and other cytokine genes in CD16+ monocytes from severe COVID-19 amplify the inflammatory response of these cells, which may contribute to disease severity.

Expression of the adhesion molecules *ITGAM* (integrin alpha M), one subunit of the β-2 integrin CD11b/CD18 or Mac-1, and *SELL* (L-selectin) is increased in severe, but not mild, COVID-19. Mac-1 and L-selectin are proteins on the surface of monocytes that mediate tethering and rolling of cells on blood vessel endothelium during transendothelial migration (43–45). During the process of monocyte transmigration, L-selectin regulates pseudopod protrusion that directs cells through interendothelial junctions with subsequent shedding of L-selectin from the surface of pseudopods (46, 47). Mac-1/CD11b, found on the lagging edge of transmigrating monocytes is bound to ICAM-1 on the endothelial surface, and cleavage of Mac-1 enables the monocyte to complete its passage through these junctions (45). Although our study did not examine protein expression levels of CD11b on CD16+ monocytes, one study showed CD11b protein is increased in total peripheral monocytes of people with COVID-19 (48). Another study reported increased CD11b protein by using flow cytometry on total monocytes from people with COVID-19 that correlated with severity of disease (49). These protein studies further support the gene expression changes we found by scRNAseq analyses. The binding of CXCL8’s to its receptors, CXCR1 and CXCR2, augments firm adhesion of monocytes to vascular endothelium, increasing transendothelial migration (50). Our results suggest that increased CXCR1, CXCR2, Mac-1/CD11b and L-selectin on CD16+ monocytes contribute to the immunopathogenesis of severe COVID-19 by increasing transmigration of these monocytes into tissues, including the lung.

Our study also found that mild cases had increased expression of genes related to cell activation and movement such as *VCAN* and *SIGLEC1*. Versican (VCAN), a proteoglycan that can be found in extracellular matrices, promotes cell adhesion, and has also been shown to stimulate inflammation by inducing cytokine release from immune cells including macrophages (51). Sialoadhesin (CD169/SIGLEC1) is a cell adhesion molecule that is expressed at low levels in monocytes and can be dramatically upregulated by type I interferon stimulation (52). A single cell study showed increased *SIGLEC1* and *VCAN* in CD14+ monocytes from COVID-19 donors compared to healthy controls (24). Our analysis showed an increase in *SIGLEC1* in CD16+ monocytes from COVID-19 cases compared to healthy cases, as well as when comparing mild cases to healthy controls. A recent study reported increased expression of sialoadhesin on total monocytes as measured by flow cytometry from people with COVID-19 compared to healthy controls (53). Another flow cytometry study reported increased CD169/SIGLEC1 protein expression on total monocytes from COVID-19 cases with mild disease compared to those with severe disease (54). Additionally, CD16+ monocytes from people with COVID-19 compared to healthy donors have increased expression of genes such as *FCGR1A* and *FCGR1B* suggesting an increase in cell activation. Although we do not report protein expression of these genes in CD16+ monocytes, another study reported that in people with COVID-19, CD64 (*FCGR1*) protein is increased in total peripheral monocytes (48), again supporting the significance of our findings.

Our study indicates that expression of genes in the S100 zinc and calcium binding protein family are increased in CD16+ monocytes in people with COVID-19 compared to healthy controls. *S100A8, S100A9* and *S100A12* are increased in both mild and severe cases, while *S100A6*, *S100A10*, and *S100A11* are increased in mild cases only. One study reported that people with severe COVID-19 have dysfunctional monocytes with high expression of S100A8/9/12 (26). S100 proteins are involved in an array of immune and inflammatory processes, including cell cycle progression, proliferation, differentiation, and cell migration (29). S100A8 and S100A9 can exist as homodimers, but are commonly found as the heterodimer, calprotectin. Calprotectin, a TLR4 ligand, is released from neutrophils, macrophages, and monocytes, and promotes secretion of multiple inflammatory proteins, including IL-6 (55). S100A9 increases the transendothelial migration of phagocytes (56). In addition, plasma levels of S100A8, S100A9, and calprotectin are increased in severe COVID-19 (21). S100A12 is produced by neutrophils, monocytes, and macrophages and mediates migration and activation of monocytes by binding to receptor for advanced glycation end products (RAGE) and TLR-4 (57, 58). Increased S100A12 is associated with rheumatoid arthritis and inflammatory bowel disease, both of which are characterized by aberrant inflammatory states (59, 60). CD16+ monocyte calprotectin secretion may contribute to increased circulating cytokines that occurs in COVID-19. Additionally, the increased expression of the calprotectin genes may further contribute to CD16+ monocyte activation and migration into tissues.

Stress conditions such as viral replication, hypoxia, or inflammation can induce cell metabolic changes. Mitochondrial metabolism in PBMC and monocytes has been shown to be altered during COVID-19 (61, 62). Our analysis of CD16+ monocytes from people with severe COVID-19 compared to those from healthy controls showed decreased expression of mitochondrial respiratory chain genes with a predicted decrease in the oxidative phosphorylation pathway. These changes were concentrated in mitochondrial complex I and complex IV. Production of ATP in monocytes can be mediated by both oxidative phosphorylation and glycolysis (63). During stress conditions or after homeostasis perturbation, transcription changes alter cellular metabolism in monocytes such that there is preferential use of one of the two ATP production pathways (64). Different types of infections or activation signals, such as hypoxia, can change the bioenergetic profile and inflammatory phenotype of monocytes (65). Microbial stimulation of different Toll-like receptors also results in changes in monocyte bioenergetics (66). In addition, mitochondrial dysfunction characterized by reduced respiration associated with reduced ATP synthase activity has been reported in peripheral blood monocytes from people with septic shock (67). Our gene expression data are consistent with findings from another study that showed by using functional and metabolic assays that in people with COVID-19, monocytes exhibit dysregulated bioenergetics characterized by decreased oxidative phosphorylation, and impaired oxidative burst (68). The diminution of gene expression related to oxidative metabolic capacity that we identified suggests that the ability of mitochondria to meet the metabolic demands of CD16+ monocytes from COVID-19 cases is compromised. Therefore, the ability of CD16+ monocytes to perform intrinsic functions that are necessary to resolve SARS-CoV-2 infection may be altered, contributing to COVID-19 disease severity.

Sustained stress conditions such as hypoxia can also induce cell death. Hypoxia is a hallmark of severe cases of COVID-19 (69). Under this condition, monocytes can activate hypoxia-inducible factor 1α (HIF-1α). We found that *HIF1A* is increased in CD16+ monocytes in severe COVID-19 cases compared to healthy controls. This is consistent with another scRNAseq study that reported an increase in *HIF1A* in total monocytes in people with COVID-19 (42). Cytosolic accumulation of HIF-1α can lead to the switch in immune cell use of oxidative phosphorylation to aerobic glycolysis. This can result in generation of excessive intracellular reactive oxygen species (ROS) due to mitochondrial dysfunction, increasing oxidative stress and subsequent cell death (70). We also found an upregulation of genes involved in oxidative stress response such as *FOS* in CD16+ monocytes from both severe and mild cases compared to healthy donors. *FOS* gene expression was reported to be increased in total monocytes in people with COVID-19 compared to healthy controls (71), similar to our results. Hypoxia can also trigger endoplasmic reticulum stress (ER), affecting protein synthesis (72). We found potent inhibition of the EIF2 pathway in CD16+ monocytes from severe COVID-19 cases, suggesting defective protein synthesis, which has been proposed to mediate apoptosis (73).

Altered interactions between monocytes and T cells may also contribute to the severity of COVID-19. HLA-DR is expressed on the surface of monocytes and is involved in antigen presentation to CD4+ T helper cells (74). Downregulation of HLA-DR on monocytes is correlated with endotoxin tolerance and reduced ability to present antigens to T cells during systemic inflammatory responses (75). Our DEG analysis showed increased levels of *HLA-DRA, HLA-DRB1* and *HLA-DRB5* in CD16+ monocytes from mild COVID-19 cases compared to healthy donors. However, in severe COVID-19 cases *HLA-DRA* and *HLA-DRB1* expression was decreased, while *HLA-DRB5* remained unaltered compared to healthy controls. A recent protein study showed that monocytes from people with severe respiratory failure due to SARS-CoV-2 infection had decreased HLA-DR, while monocyte HLA-DR expression in people presenting with milder COVID-19 was similar to that of healthy controls (40). Another report showed a predominance of HLA-DR^low^ monocytes from people with severe COVID-19, suggesting low HLA-DR may contribute to a dysfunctional immune response (26). Two studies showed by flow cytometry that the numbers of CD16+ monocytes were lower in the peripheral blood of individuals with severe COVID-19 compared to mild and control groups, and that within this population, there was decreased HLA-DR expression (21, 41). Although we did not examine HLA-DR protein expression in this study, HLA-DR cell surface protein expression was reported to be decreased in CD14+ and CD16+ monocytes in people with COVID-19, with decreased expression correlating with disease severity (21, 26, 40, 48, 76). Additionally, a flow cytometry study showed that protein expression of HLA-DR and CD86 was found to be decreased, and CD163 increased on CD14+ and CD16+ monocytes in severe cases of COVID-19 (77). In our analysis, *CD86* gene expression decreases and *CD163* increases in CD16+ monocytes in severe cases of COVID-19 compared to healthy controls. Decreased CD4 protein expression on total peripheral monocytes in people with COVID-19 correlates with disease severity as measured by flow cytometry (78). In our analysis of CD16+ monocytes, *CD4* gene expression is significantly reduced in people with severe, but not mild, disease compared to healthy controls. The experimental results of the above-mentioned protein studies are consistent with our gene expression changes. These findings suggest defective interactions between CD4+ T helper cells and CD16+ monocytes in people with severe COVID-19. This may contribute to an impaired immune response against SARS-CoV-2, resulting in severe disease manifestations.

Our study highlights the potential contribution of CD16+ monocytes to the pathogenesis of SARS-CoV-2. Although CD16+ monocytes are not productively infected with the virus, they showed significant transcriptional changes compared to cells from healthy individuals. These changes may contribute to an inflammatory response in an infected individual, including induction of a strong antiviral interferon response and production of cytokines and chemokines. Additionally, these cells have a migratory phenotype suggestive of their increased recruitment into tissues where they can contribute to an inflammatory cascade. SARS-CoV-2 infection of individuals may also contribute to dysregulation in CD16+ cell metabolism and HLA-DR expression, and increased apoptosis. Our findings are supported by recent studies showing protein dysregulation of HLA genes and functional metabolic changes in CD16+ monocytes from people with COVID-19. These changes may impact the function of CD16+ monocytes, including reduced antigen presentation, contributing to disease severity.

There are several limitations in our study. We were dependent on the disease severity classification established in the reports from which our samples were obtained. Hence, the parameters to classify disease severity may be inconsistent among data sets. Another limitation is the lack of longitudinal samples collected for many of the datasets. Due to this, we were not able to characterize gene profiles in individuals throughout their disease course. This study also does not address the effects of age, race and ethnicity, comorbidities, or treatment regimens, which were not always available from the datasets. For this study, we applied QC metrics to discard cells expressing more than 15-25% of mitochondrial genes. This limitation may have excluded from the analysis the cells that had high mitochondrial expression as a consequence of SARS-CoV-2 infection of the individual. Another limitation is the low numbers of donors that we were able to include in our analysis. Additionally, changes in gene expression do not always correlate with protein level changes, so proteomic studies are needed to confirm the differential gene expression identified in CD16+ monocytes from people with COVID-19 in this study.

## Data Availability Statement

Publicly available datasets were analyzed in this study. This data can be found here: Gene Expression Omnibus (GEO) database with the accession numbers GSE150728, GSE158055 and GSE149689, and the European Genome-phenome Archive (EGA) under access number EGAS00001004571. We also included a publicly available healthy control sample downloaded from the 10X Genomics official website (https://support.10xgenomics.com/single-cell-gene-expression/datasets/3.1.0/5k_pbmc_NGSC3_aggr).

## Author Contributions

JB, PM-A, and VC designed this study. PM-A and VC performed the data analysis. JB, LX, PM-A, and VC contributed to the discussions about computational analysis. PM-A, TC and VC wrote the manuscript. AM, JB, LC, LX, PM-A, TC, VC, VR, and VV contributed to discussions about manuscript content, writing, and editing of the manuscript. All authors contributed to the article and approved the submitted version.

## Funding

This work was funded by R01DA041931 (TC, JB), R01DA044584 (VC, LC, JB), R01DA048609 (LX, LC, JB), R01MH112391 (PM-A, TC, JB), T32AI007501(VR), T32GM007288 (VR), TL1TR002557 (AM), Innovation in Cancer Informatics Fund (LX) and Burroughs Wellcome Fund 342478 (VV).

## Conflict of Interest

The authors declare that the research was conducted in the absence of any commercial or financial relationships that could be construed as a potential conflict of interest.
